# A genetic screen in *Drosophila* uncovers a role for *senseless-2* in surface glia in the peripheral nervous system to regulate CNS morphology

**DOI:** 10.1093/g3journal/jkae152

**Published:** 2024-07-12

**Authors:** Haluk Lacin, Yuqing Zhu, Jose T DiPaola, Beth A Wilson, Yi Zhu, James B Skeath

**Affiliations:** Division of Biological and Biomedical Systems, University of Missouri-Kansas City, 5009 Rockhill Road, Kansas City, MO 64110, USA; Department of Genetics, Washington University School of Medicine, 4523 Clayton Avenue, St. Louis, MO 63110, USA; Department of Genetics, Washington University School of Medicine, 4523 Clayton Avenue, St. Louis, MO 63110, USA; Department of Genetics, Washington University School of Medicine, 4523 Clayton Avenue, St. Louis, MO 63110, USA; Department of Genetics, Washington University School of Medicine, 4523 Clayton Avenue, St. Louis, MO 63110, USA; Department of Genetics, Washington University School of Medicine, 4523 Clayton Avenue, St. Louis, MO 63110, USA

**Keywords:** *Drosophila*, *senseless-2*, *papilin*, CNS, glia, basement membrane

## Abstract

Despite increasing in mass approximately 100-fold during larval life, the *Drosophila* CNS maintains its characteristic form. Dynamic interactions between the overlying basement membrane and underlying surface glia are known to regulate CNS structure in *Drosophila*, but the genes and pathways that establish and maintain CNS morphology during development remain poorly characterized. To identify genes that regulate CNS shape in *Drosophila*, we conducted an EMS-based, forward genetic screen of the second chromosome, uncovered 50 mutations that disrupt CNS structure, and mapped these alleles to 17 genes. Analysis of whole genome sequencing data wedded to genetic studies uncovered the affected gene for all but 1 mutation. Identified genes include well-characterized regulators of tissue shape, like *LanB1*, *viking*, and *Collagen type IV alpha1*, and previously characterized genes, such as *Toll-2* and *Rme-8*, with no known role in regulating CNS structure. We also uncovered that *papilin* and *C1GalTA* likely act in the same pathway to regulate CNS structure and found that the fly homolog of a glucuronosyltransferase, B4GAT1/LARGE1, that regulates Dystroglycan function in mammals is required to maintain CNS shape in *Drosophila*. Finally, we show that the *senseless-2* transcription factor is expressed and functions specifically in surface glia found on peripheral nerves but not in the CNS to govern CNS structure, identifying a gene that functionally subdivides a glial subtype along the peripheral–central axis. Future work on these genes should clarify the genetic mechanisms that ensure the homeostasis of CNS form during development.

## Teaser

This paper describes a genetic screen that identified 17 genes that regulate CNS morphology in *Drosophila*. Genetic approaches together with whole genome sequencing analysis uncovered the molecular nature and causative lesions for 16 of these genes. Characterization of one gene, *senseless-2*, which encodes a zinc finger transcription factor, revealed that it is expressed and functions in surface glia in the peripheral nervous system (PNS), but not CNS, to govern CNS structure, identifying one of the first genes known to subdivide a glial subtype along the PNS-CNS axis.

## Introduction

Structure determines function is a unifying theme of biology. A thorough understanding of the genes that govern tissue or organ structure is, however, lacking. We use the *Drosophila* larval CNS as a model system to investigate the genetic factors that control tissue shape (Skeath *et al*. 2017). In response to the concerted action of hemocytes, neural activity, and glial cell function in a process called nerve cord condensation, the *Drosophila* larval CNS adopts its distinctive shape at the end of embryogenesis upon the shortening and thickening of the nerve cord (Olofsson and Page 2005; Karkali *et al*. 2022). Once its shape is established, the *Drosophila* CNS maintains its form throughout its massive growth during larval development.

The *Drosophila* larval CNS is consecutively and fully enwrapped by the CNS basement membrane and the perineurial and subperineurial surface glial cells (Stork *et al*. 2008; Meyer *et al*. 2014; Yildirim *et al*. 2019). The basement membrane is composed of independent Collagen IV and Laminin networks that are connected to each other by Perlecan; both networks establish connections to surface glial cells through interactions with the Integrin and Dystroglycan cell surface receptors (Yurchenco 2011). The tethering of the basement membrane to surface glial cells provides the CNS with structural and biomechanical strength essential to maintain its distinct shape. Interactions between the basement membrane and surface glia are, however, far from static. The tremendous growth of the CNS during larval life demands a dynamic basement membrane-surface glia interface that imparts structure to the CNS, while being continually remodeled to allow tissue growth and expansion.

In the larvae, the fat body secretes basement membrane proteins that are continuously deposited on the CNS basement membrane during development (Pastor-Pareja and Xu 2011), coordinating basement membrane deposition with tissue growth in the CNS. Remodeling of the basement membrane occurs at least in part through the action of the membrane-tethered and secreted extracellular proteases MMP1, MMP2, and AdamTS-A, which are expressed by surface glia (Page-McCaw *et al*. 2003; Meyer *et al*. 2014; Skeath *et al*. 2017). These proteases oppose the tissue stiffening action of Collagen IV by cleaving basement membrane proteins and in so doing help maintain a delicate balance between tissue stiffness and softness that maintains tissue structure while allowing tissue growth and expansion (Apte 2009; Miller *et al*. 2011; Rodriguez-Manzaneque *et al*. 2015; Skeath *et al*. 2017). In addition to the fat body, migratory hemocytes also express basement membrane proteins in larvae and may serve as a second source of basement membrane proteins and a basement membrane remodeling agent (Isabella and Horne-Badovinac 2015).

To gain more insight into the factors that regulate CNS shape, we undertook a large-scale forward genetic screen of the second chromosome to identify mutations that disrupt CNS structure. Our screen identified 17 genes, including characterized regulators of tissue structure, characterized genes with no known role in controlling tissue structure, such as *Toll-2* (*18-wheeler*) and *Rme-8*, and previously uncharacterized genes, like a glucuronosyltransferase homologous to B4GAT1 and LARGE1/2, which regulate Dystroglycan in mammals, and the transcription factor Senseless-2. Our results highlight an unexpected role for perineurial glia in the peripheral nervous system (PNS) to help to establish and maintain CNS structure.

## Materials and Methods

**Fig. 1.**
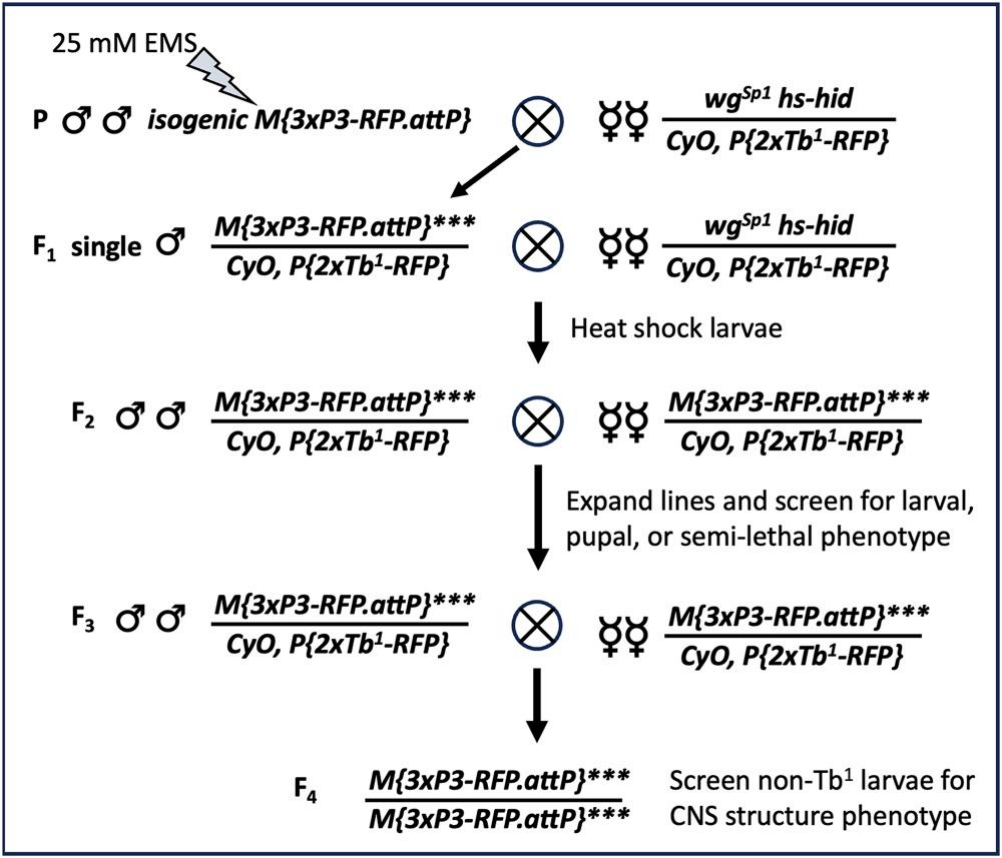
Diagram of the genetic cross scheme used to execute the forward genetic screen.

Genetic screen: To identify mutations that disrupt larval CNS morphology, we performed a standard, autosomal recessive forward genetic screen of the second chromosome using a chromosome isogenic for the *M[3xP3-RFP.attP]* ϕC31 “landing pad” transgene inserted in cytological location 51D. The *3xP3-RFP* construct in this transgene expresses RFP in most glia and allows rapid visualization of CNS morphology in live larvae (Zhu *et al*. 2024). As outlined in Fig. 1, we mutagenized isogenic *M[3xP3-RFP.attP]* males with 25 mM EMS for 8 h, allowed them to recover from EMS treatment overnight, and then mated them *en masse* to *CyO P[Tb^1^-RFP]/wg^Sp1^ P[Hs-hid]* virgin females, discarding mutagenized males on day 5. The CyO *P[Tb^1^-RFP]* chromosome carries the larval marker *Tubby* (*Tb*), facilitating the identification of larvae that carry or do not carry this balancer based on larval body shape. The *wg^Sp1^ P[Hs-hid]* chromosome carries the *hid* transgene under the control of the *hsp70* promoter, which induces activation of the proapoptotic *hid* gene upon heatshock, killing all flies that carry this transgene. 17,790 F1 males of the M[3xP3-RFP.attP]***/CyO *P[Tb^1^-RFP]* genotype, where “***” denotes the mutagenized chromosome, were individually crossed to 3–5 *CyO P[Tb^1^-RFP]/wg^Sp1^ P[Hs-hid]* virgin females and allowed to mate for 7 days before adults were discarded. On days 8 and 9, each vial was heat-shocked for 30–35 min at 37°C to ensure that only F2 progeny of the following genotype survived: M[3xP3-RFP.attP]***/CyO *P[Tb^1^-RFP]*; 12,211 of the 17,790 single male crosses were successful; for each of these crosses, F2 progeny were sib-mated to expand the stock and resulting F3 progeny were screened for larval-, pupal-, or semilethal phenotypes; 3,180 lines displayed such a phenotype. For each of these lines, F4 larvae homozygous for the mutagenized chromosome, easily identified by their lack of the *Tb* marker, were visually screened for disrupted CNS morphology, e.g. an elongated CNS, under a fluorescent dissecting microscope. Fifty lines were identified that harbored second chromosomal mutations that when homozygous disrupted the morphology of the larval CNS.

Complementation analysis: The 50 mutations were grouped into 3 phenotypic classes: those with an elongated CNS (*n* = 34), a herniated or misshapen CNS (*n* = 15), or a wider CNS (*n* = 1). Exhaustive complementation crosses among lines within each group identified 9 complementation groups and 9 “singleton” mutations. Crosses of each of these lines to second chromosomal mutations known to disrupt CNS morphology, such as *Laminin B1 (LanB1)* and the tightly linked *viking* and *Collagen Type IV alpha1 (Col4a1)* genes, which are uncovered by the deficiency, *Df(2L)BSC172*, identified 9 alleles of *LanB1* and one each of *viking* and *Col4a1*, leaving 8 complementation groups and 6 singletons.

Whole genome sequencing: At least 3 alleles per complementation group, all singleton mutations, and the isogenic target chromosome were subjected to whole genome sequencing. For each allele, 10–15 late-third instar larvae homozygous for the relevant mutagenized M[3xP3-RFP.attP]*** chromosomes were collected, washed, and frozen. Genomic DNA extractions were performed using the Qiagen DNeasy Blood & Tissue Kit and and Illumina Nextera Flex DNA Library Prep Kit was used to prepare indexed next-generation sequencing libraries following the manufacturer's protocol. Genomic DNA was then provided to GTAC (Washington University) for whole genome sequencing at ≥30× coverage. Sequence reads were mapped to *Drosophila* reference BDGP6 using NovoAlign (Novocraft Technologies). Sentieon software was used to remove duplicate reads, realign reads around indels, recalibrate quality scores, and call variants. The Sentieon DNAscope tool was used to identify variants. Joint variant calls were made using the mutant lines and a reference line. Variants were annotated using SnpEff and the variants were filtered using SnpSift to select unique coding variants within previously determined genomic intervals. Ultimately, this process identified nucleotide differences or variants in coding regions and splice sites and small deletions that differed between the mutagenized chromosome and the isogenic target chromosome; these variants are likely EMS-induced mutations. On average, our sequencing and bioinformatics approach identified 19.4 likely EMS-induced mutations in coding regions or splice sites per mutagenized second chromosome (range 7–32), using a minimum read depth of 20 as a cutoff and requiring homozygosity or near homozygosity for the variant. For singletons, this approach identified a list of putative EMS-induced mutations, one of which is likely the causative lesion. For complementation groups, this approach identified the likely causative lesion for all complementation groups, as only one gene in each complementation group harbored independent EMS-induced mutations in its coding region in all sequenced alleles. When possible, we confirmed correspondence between gene and mutant phenotype via complementation crosses with appropriate deficiencies and gene-specific alleles and phenotypic analysis. All putative causative lesions were confirmed via PCR-based methods and Sanger sequencing (GeneWiz Inc.).

Genbank accession information for whole genome sequence information: BioProject ID PRJNA1128589.

Genetic mapping and complementation crosses: To identify the causative lesion in singleton alleles, we took 2 approaches. For 3 lines, we used complementation crosses against deficiencies or gene-specific alleles that uncovered mutations likely to cause the associated CNS phenotype to confirm the correspondence between gene and mutant phenotype. This approach identified mutations in *diaphanous*, *pvf3*, and *lethal giant larvae* as the causative to the CNS phenotype. For the remaining 3 singletons, we used the approach of Zhai *et al*. (2003) to map the causative lesion in each line on the genetic map relative to 4 P[w+] element inserts located at genetic map positions 13, 52, 62, and 86 in the second chromosome. Using this approach, we mapped 1 singleton to genetic map positions 18–23 and 53–57 in the second chromosome; the results for the third singleton were inconclusive likely due to the presence of 2 or more lethal mutations in the chromosome. Complementation crosses against appropriate deficiencies and gene-specific alleles followed by phenotypic analysis identified *Tango1* as the singleton on 2L and *worniu* as the singleton near genetic map positions 53–57. The 2 putative causative lesions were confirmed by PCR-based methods and Sanger sequencing.

Database/knowledgebase usage: We used FlyBase (release FB2024_02) to find information on fly lines, mutant alleles, protein structure, etc. (Ozturk-Colak *et al*. 2024).

Transgene construction: The *UAS-senseless-2* transgene was generated by amplifying nucleotides 276–2,529 of the *sens-2 cDNA*, FI 20031 (accession number BT33368). The resulting PCR product was cloned into NotI and KpnI sites of pUAS-T. DNA was coinjected with helper plasmid into embryos of the genotype w1118 by Rainbow Transgenic Flies, Inc.

Due to the large size of *ppn*, the UAS-Ppn[RG] transgene was constructed piecemeal through PCR-based approaches amplified defined regions of partial cDNA clones and genomic DNA; these individual regions were then stitched together via Gibson cloning. First, we used the GH25513 cDNA clone, which contained a portion of the 5′ coding region of Ppn (accession number BT100225) to amplify nucleotides 1–2,471 of the coding region of the Ppn[RG] transcript starting with the ATG start codon in exon 1 and continuing into exon 6. Second, we used genomic DNA from a strain of flies isogenic for a wild-type third chromosome to amplify nucleotides 2,452–5,205 within exon 6 nucleotide 5,162–5,384 (exons 7) of the Ppn[RG] transcript. Third, we used the partial 3′ GH05059 cDNA clone (accession number AY060635) to amplify in separate reactions nucleotides 5,344–6,602 (exons 8–9) and nucleotides 6,556–8,526 (exons 10–17, including *the* ATT stop-codon) of the Ppn[RG] transcript. The PCR products were assembled via Gibson cloning and inserted into the KpnI and XbaI sites of the phiC-based construct pJFRC28, which was then injected into the P[CaryP] attp2 fly line (BDSC: 8622) with phiC integrase by Rainbow Transgenic Flies, Inc.

### Gene rescue and in vivo RNAi phenocopy assays

To restrict UAS-linked transgene expression specifically to glia, we used the *repo-GAL4* driver line. To restrict UAS-linked transgene expression specifically to neurons, we paired the *elav-GAL4* driver line, which activates transgene expression strongly in all neurons and moderately in glia, with *repo-GAL80*, which blocks GAL4-dependent activation in glia (Awasaki *et al*. 2008). Gene rescue experiments were performed in the *sens-2-T2A-GAL4/sens-2^js4^* background.

Allele nomenclature: Alleles were given with their final designation (e.g. js1, js2) after they were placed in complementation groups. Within each complementation group alleles were named based on the order in which they were identified in the screen. The 1 singleton mutation was designated *jsz4*.

Gene expression analysis: Gene expression analysis was performed essentially as described in Patel (1994). Briefly, the larval CNS was dissected in PBS, fixed in 3.7% formaldehyde for 35 min, and washed in PTx (1×PBS, 0.1% TritonX-100) 5 times over 1 h prior to primary antibody incubation. Fixed tissues were incubated in primary antibody with gentle rocking overnight at room temperature, then washed 5 times in PTx over the course of an hour, incubated in the appropriate secondary overnight for at least 4 h to overnight at room temperature, and then washed again in PTx at least 5 times over 1 h. The CNS was then dissected and mounted in PTx or in DPX mountant after dehydration via an ethanol series and clearing in xylenes (Truman *et al*. 1994). All imaging was performed on a Zeiss LSM-700 Confocal Microscope, using Zen software. The following antibodies were used in the study: Deadpan (1:2,000; Skeath *et al*. 2017); rat monoclonal antibody 7E8A10 (ELAV; 1:200; O'Neill *et al*. 1994); mouse monoclonal antibody 8D12 (REPO; 1:100) (Alfonso and Jones 2002); rabbit anti-Sens-2 (this paper; 1:500); rabbit anti-Ppn (this paper; 1:250); goat Anti-GFP Dylight TM 488 Conjugated, preadsorbed (Rockland; #600-141-215). Secondary antibodies were obtained from the following sources. The Alexa Fluor Plus 488 secondary antibodies of appropriate species specificity were obtained from ThermoFisher and used at a dilution of 1:400: e.g. Donkey anti-Rat IgG Alexa Fluor Plus 488; catalog number Cat #A48269. The Cy5 secondary antibodies of appropriate species specificity were obtained from Jackson Immunoresearch and used at a dilution of 1:400: e.g. Cy5 Donkey Anti-Rat IgG, #712-175-153. Please see the Supplementary, Reagents Table for a complete description of secondary antibodies used in this study.

### Antibody generation

To generate the *Senseless-2* antibody, DNA encoding amino acids 1–199 of Senseless-2 was inserted into pET-29a (Novagen) for protein expression and purification. Protein-specific antibody responses were mounted in rabbits (Pocono Rabbit Farm and Laboratory, PA, USA) and the resulting sera were used at a 1:500–1:1,000 dilution. As all senseless-2 mutations arise C-terminal to the epitopes against which the antibody was generated, we confirmed the specificity of this antibody to senseless-2 by driving UAS-senseless-2 expression in subperineural glia within the ventral nerve cord, which normally do not express *senseless-2*, and detecting high levels of Senseless-2 protein in these cells (Supplementary Fig. 1).

Papilin antibody generation: YenZym (CA, USA) was used as a commercial source to generate affinity-purified antibodies against 2 distinct synthetic peptides that corresponded to amino acids 27–47 (RFPGLRQKRQYGANMYLPEC) and 2,670–2,691 (TRPVTQRPSYPYRPTRPAYVPE) of Ppn-PG. Briefly, each peptide was conjugated to KLH, used as an immunogen in rabbits to generate a peptide-specific antibody response, and antibodies specific to the peptide were affinity purified. The 2 affinity-purified antibodies were combined and used at a dilution of 1:250–1:500 for immunofluorescence analysis.

### Generation of senseless-2 CRIMIC T2A-GAL4 line

To generate sens-2-GAL4-DBD, we used a modified version of the strategy developed by Kanca *et al*. (2019). We used the Genewiz company to synthesize a DNA fragment into the EcoRV site of the pUC-GW-Kan vector. This fragment is made of the left and right homology arms (HA) which are immediately adjacent to the cut site and restriction enzyme sites (SacI-KpnI) between these arms. We then directionally cloned the attp-FRT-splitGAL4-FRT-attp fragment (see below) into the middle of left and right HAs using the SacI and KpnI sites. We used the genome sequence of the injected flies (vasa-cas9, BDSC-51324) for design.

*HA flanking sequences:*

Left HA: 5′cgtgtgtgagagaga 3′aacggtcttttccct;

Right HA: 5′gggtggggcagcgcc 3′tatctatcatagata

*SacI-attp-FRT-splitGAL4-FRT-attp-Kpn1 fragment:*

We used the Gibson cloning method to clone 3 fragments into pBS-KS digested with SacI and KpnI. Primer pairs and templates are shown below: Note we also included ECoRI and EcoRV sites for replacing the trojan exon between attp-FRT sites as a back-up strategy when needed.

1. *SacI-*attp-FRT from pM14 (Kanca *et al*. 2019):
  - F actcactatagggcgaattgGAGCTC*acggacacaccgaag*
  - R *caagtcgccatgttggatcgac*
2. Split-GAL4 from Trojan AD/DBD (Diao *et al*. 2015):
  - F cta*gaaagtataggaacttc*GAATTC**agtcgatccaacatggcgacttg**
  - R cttt*ctagagaataggaacttc*GATATC**aaacgagtttttaagcaaactcactcc**
3. Kpn1-attp-FRT from pM14 (Kanca *et al*. 2019):
  - F **ggagtgagtttgcttaaaaactcgttt**GATATCgaag*ttcctattctctagaaag*
  - R cactaaagggaacaaaagctgGGTACCgtac*tgacggacacaccgaag*

Corresponding sequences from pBS-KS are underlined, pM14 are in italics, and Trojan AD/DBD are in bold; restriction enzyme sites are in all caps.

Guide RNAs were identified with CRISPR target Finder (Gratz *et al*. 2014) with maximum stringency and minimal off-target effects. We used pCFD4 vector (Port *et al*. 2014) to clone *sens-2* specific guide and donor linearizing guide RNAs via a PCR intermediate with the following primers:

*sens-2 guide RNA*: attttaacttgctatttctagctctaaaacCCCAACGGTCTTTTCCCTGC gacgttaaattgaaaataggtc

*linearizing guide RNA*: tatataggaaagatatccgggtgaacttcGTAGTACGATCATAACAACG gttttagagctagaaatagcaag

All constructs were injected into *vasa-cas9* (BDSC #51324), which were then crossed to *Tubulin-GAL4-AD*, *UAS-TdTomato/CyO,* and scored for TdTomato expression to identify positive lines. Verification of targeting was confirmed via PCR-based sequencing methods. After we generated the *sens-2-GAL4-DBD* line, we used the strategy described in Diao *et al*. (2015) to replace the split-GAL4 insert with a T2A-GAL4 and then conducted all experiments with the sens-2-T2A-GAL4 line, termed *sens-2-GAL4*.

The *papilin*-T2A-GAL4 line was generated from the *ppn^MI03189^* insert following the genetic cross scheme outlined in Diao *et al*. (2015).

Fly strains: Please see Supplementary Table 1 in Supplementary File 1 and Reagents Table (Supplementary File 2) for a complete list of fly stocks used in this paper.

## Results and discussion

**Fig. 2.**
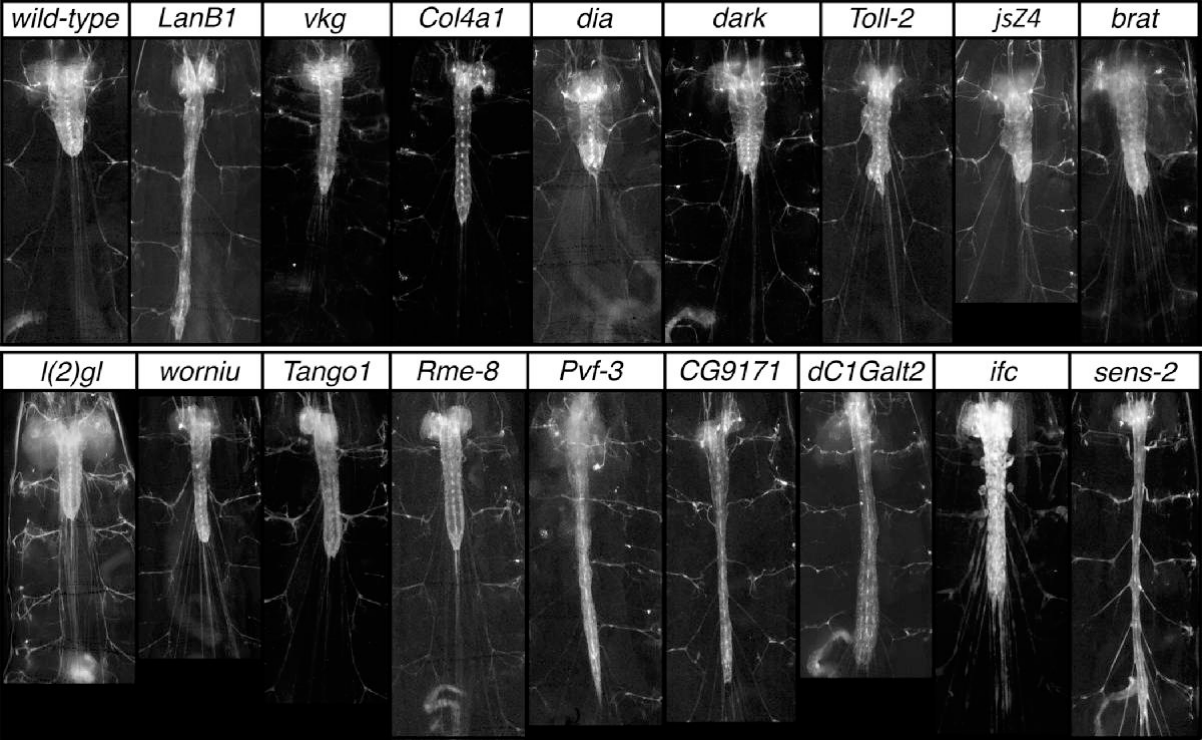
Representative examples of CNS mutant phenotypes were identified in the genetic screen. Ventral views of wild-type and mutant late-third instar larvae of the indicated genotype showing 3xP3 RFP staining in grayscale to highlight CNS morphology. Anterior is up; scale bar is 500 μm.

The cellular and molecular processes that control CNS shape are poorly understood. To gain insight into these processes, we performed a large-scale EMS-based, forward genetic screen of the second chromosome to identify mutations that disrupt the morphology of the larval CNS (Fig. 1). Briefly, we used a target chromosome that contains a *3xP3-RFP* transgene insert in the second chromosome that labels most glia (Zhu *et al*. 2024), enabling rapid visualization of the CNS in live larvae. We set up 17,790 single male F1 crosses, screened 12,211 F3 lines for second chromosomal mutations that when homozygous were larval-, pupal-, or semilethal, identified 3,180 such lines, and visually screened each for defects in overall CNS morphology. We identified 50 mutations that disrupt CNS shape, including 11 mutations in *LanB1*, *viking*, or *Col4a1*, genes which have well-characterized roles in regulating tissue and CNS structure (Pastor-Pareja and Xu 2011; Kim *et al*. 2014; Skeath *et al*. 2017; Dai *et al*. 2018). The 39 remaining mutations were grouped into 3 phenotypic classes: those with a wider CNS (*n* = 1), a herniated or misshapen CNS (*n* = 15), and an elongated CNS (*n* = 23). Complementation crosses identified 8 complementation groups and 6 “singleton” mutations among these 39 lines, and analysis of whole genome sequencing data of most lines at ≥30× coverage identified the causative lesion(s) in and affected gene for 13 of the 14 genes. When possible, phenotypic analysis with appropriate deficiencies and gene-specific alleles was used to confirm correspondence between gene and mutant phenotype. The CNS phenotype, molecular nature, and known function of these genes are summarized in Table 1 and Fig. 2.

**Table 1.** Molecular nature, phenotypic class, and causative lesions of identified genes.

Gene (alleles)	CNS phenotype	Causative mutations	Protein function
*LanB1* (9)	Highly elongated	Not sequenced	Basement membrane protein
*viking* (1)	Elongated	*vkg^js1^*: G585D	Basement membrane protein
*Collagen 4a1* (1)	Elongated	*Col4a1^js1^:* G1702S	Basement membrane protein
*diaphanous* (1)	Wide	*dia^js1^*: A51*fs*	Dia-class formin
*Toll-2 (18-wheeler)* (3)	Misshapen	*18w^js1^*: N293H *18w^js2^*: Q477* 18w^js3^: Q90*	Toll-like receptor family (Toll-2)
*dark* (11)	Misshapen (mild)	*Dark^js1−js11^*: Identical 370 bp that deletes amino acids 830–952 and introduces a frameshift after the deletion	Essential component of apoptosome
*jsZ4* (*1*)	Misshapen	Unknown	Unknown
*brat* (3)	Elongated	*brat^js1^*: 114 bp deletion that removes start codon *brat^js2^*: W688* brat^js3^: Q294*	Tumor suppressor
*Lethal(2) giant larvae* (1)	Elongated	*lgl^js1^*: T331*fs*	Tumor suppressor
worniu (1)	Elongated	*wor^js1^*: L117*	Zinc finger C2H2 transcription factor
*Tango1* (1)	Elongated	*Tango1^js1^*: T60I	Protein secretion
*Rme-8* (3)	Elongated	*Rme-8^js1^*: W837* Rme-8^js2^: P1200S *Rme-8^js3^*: K1779-Splice Acceptor mutation (c.5337 − 1C > T, intron 15)	DnaJ domain-containing Hsp40 family protein involved in receptor-mediated endocytosis
*Pvf3* (1)	Highly elongated	*Pvf3^js1^*: A451-splice donor mutation (c.1351 + 1C > T, intron 2)	PDGF- and VEGF-related ligand for Pvr receptor
*CG9171* (4)	Highly elongated	*CG9171^js1^*: I240N *CG9171^js2^*: T214I *CG9171^js3^*: R336-splice donor mutation (c.1127 + 1C > T, intron 3) *CG9171^js4^*: E489V	Glucuronosyltransferase
*dC1GalTA* (2)	Highly elongated	*dC1GalTA^js1^*: K298* dC1GalTA^js2^: M191K	Core 1 Galactosyltransferase A
*infertile crescent* (3)	Highly elongated	*ifc^js1^*: V276D *ifc^js2^*: G257S *ifc^js3^*: W240*	Dihydroceramide desaturase
*senseless*-*2* (4)	Highly elongated	*sens-2^js1^*: R590L *sens-2^js2^*: G421-splice acceptor mutation (c. 1263–1G > A, intron 2) *sens-2^js3^*: C622Y *sens-2^js4^*: R258*fs*	Zinc finger C2H2 transcription factor

Below, we outline the genes identified in the screen and their associated mutations and CNS phenotypes, starting with the gene/mutation that yielded a wider CNS and ending with those that yielded an elongated CNS. We describe in greater detail the function of the *senseless-2* gene, which encodes a Zinc finger transcription factor that may act as a genetic switch to distinguish perineurial glial found in the PNS from those in the CNS.

### Gene that yields a widened CNS phenotype when mutated

**Fig. 3.**
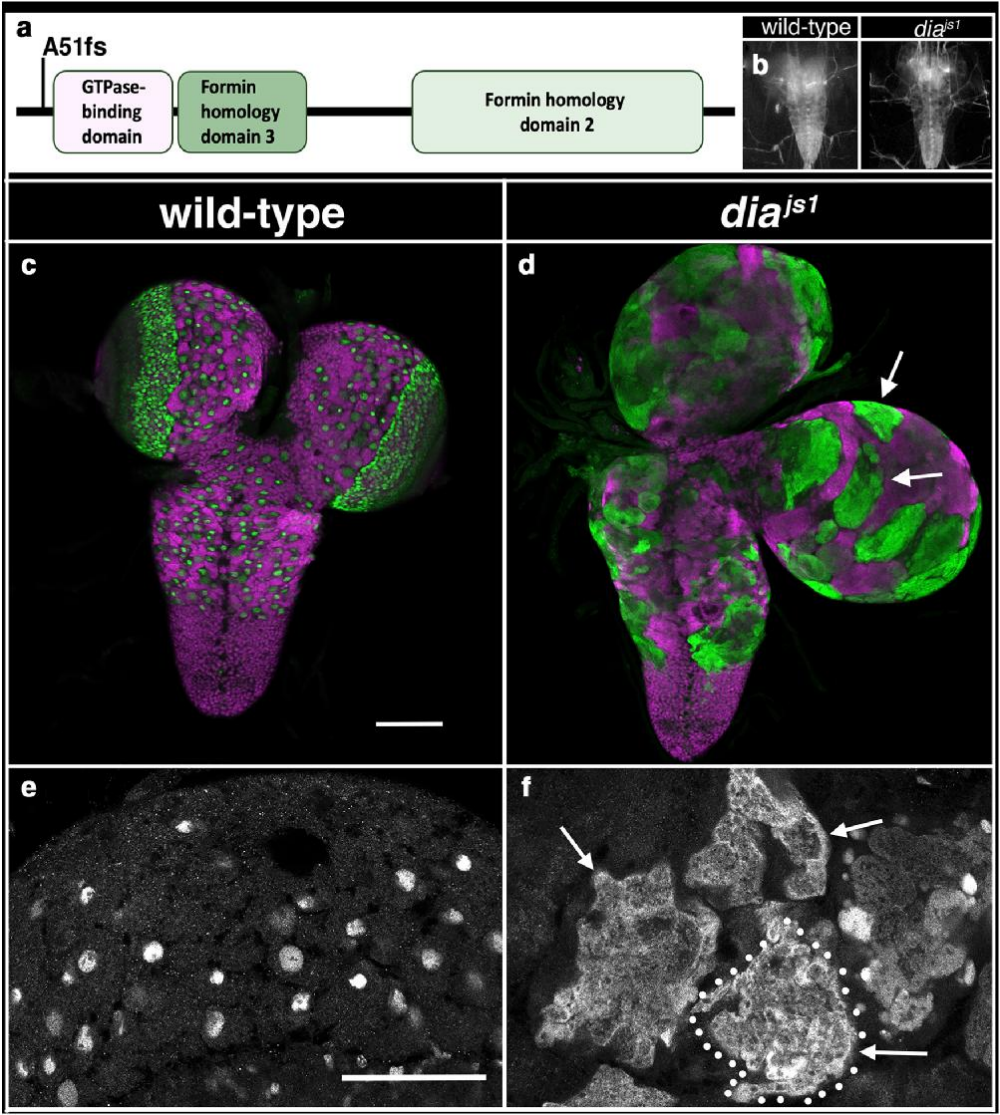
Mutations in *diaphanous* increase the width of the CNS. a) Schematic of Diaphanous protein with location and nature of the *dia^js1^* allele. b) Ventral views of wild-type or *dia^js1^* mutant late-third instar larvae with 3xP3 RFP expression (grayscale) highlighting the CNS. Anterior is up; scale bar is 250 μm. c–f) Ventral views of dissected CNS (c, d) or high magnification view of the brain (e, f) from wild-type and *dia^js1^* mutant late-third instar larvae labeled for neuroblasts with Deadpan (Dpn is green in panels c and d and gray in panels e and f) and for neurons with ELAV (magenta in c, d). Arrows highlight enlarged neuroblasts; dotted lines demarcate the presumed boundary of 1 enlarged neuroblast. Anterior is up; scale bar is 100 μm in panels c and d and 50 μm in panels e and f.

*diaphanous*: We identified 1 mutant allele that when homozygous yielded a wider CNS. This allele identified a frameshift mutation found immediately after the codon for amino acid 51 in the *diaphanous* coding region. Larvae homozygous for this allele or trans-heterozygous for it and *dia^5^* or *Df(2L)ED1315*, a deficiency of the region, resulted in an expansion of the thoracic region of the CNS that became more pronounced as larvae were about to pupariate (Table 1; Figs. 2 and 3b). *diaphanous* encodes the sole Dia-class formin protein in *Drosophila*; it is required for cytokinesis and induces polyploidy in follicle cells and mitotic neuroblasts (Castrillon and Wasserman 1994). Using antibodies specific to Deadpan and ELAV to label neuroblasts and neurons, respectively, we observed a massive increase in neuroblast size likely caused by defects in cytokinesis and increased ploidy of these cells (Fig. 3c–f). Our results indicate that the expanded nature of the thoracic ventral nerve cord in *diaphanous* mutants likely results from the expanded size of neuroblasts and potentially other cell types in the CNS.

### Genes that yield a misshapen CNS phenotype when mutated

We identified mutations in 3 genes—*Dark*, *Toll-2*, and 1 singleton mutation—that gave rise to a misshapen CNS (Table 1). We identified 11 alleles of *Dark*. Homozygosity for each allele or trans-heterozygosity for most combinations of these alleles yielded a mild, partially penetrant CNS phenotype in which small hernias arise in the CNS (Fig. 2). This phenotype was also recapitulated when each allele was placed over a deficiency of the region, *Df(3R)BSC331*. We sequenced the 11 alleles, each of which contained an apparently identical 370-bp deletion within the coding region (Table 1). The identity of the molecular lesion in the alleles suggests the mutations arose in mitotic spermatagonia or spermatocyte cells and are not independent of each other. *Dark* encodes a homolog of the Apaf-1/Ced-4 proapoptotic genes and has been shown to promote hyperplasia in the CNS (Rodriguez *et al*. 1999).

**Fig. 4.**
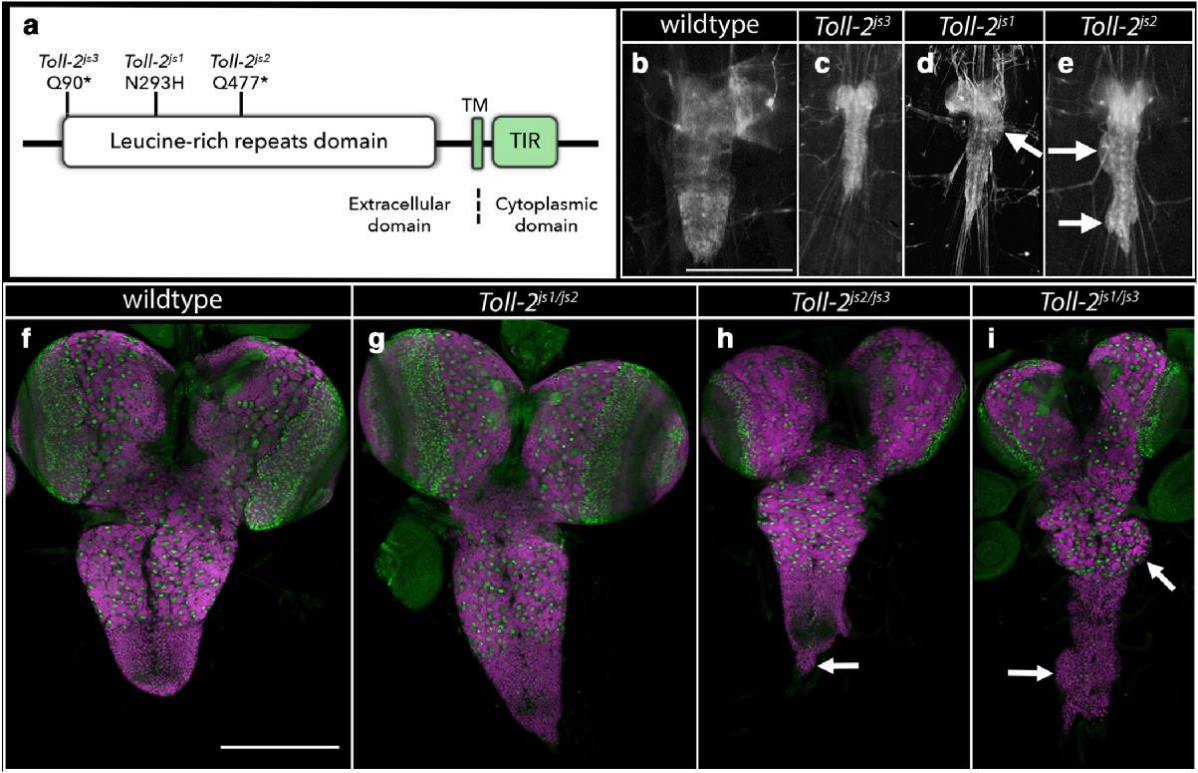
Loss of *Toll-2* function results in a misshapen CNS. a) Schematic of Toll-2 protein with location and nature of identified *Toll-2* alleles. b–e) Ventral views of wild-type or *Toll-2* mutant late-third instar larvae in which 3xP3 RFP expression highlights the CNS. Arrows highlight hernias, bulges, or dysmorphology in the CNS. Anterior is up; scale bar is 500μm. f–i) Ventral views of dissected CNS of wild-type (f) and *Toll-2* mutant (g–i) late-third instar larvae labeled for neuroblasts with Deadpan (Dpn; green) and neurons with ELAV (magenta). Arrows point to areas of the CNS that display dysmorphology. Anterior is up; scale bar is 200 μm.

*Toll-2*: Three allelic mutations identified the *Toll-2* (*18-wheeler*) gene with 2 of the mutations being premature stop codons and the third being a missense mutation in the leucine-rich repeat domain (Table 1; Fig. 4a). Homozygosity for each allele or trans-heterozygosity for all combinations of these alleles yielded a phenotype in which the CNS was reduced in size and the ventral nerved cord was misshapen and marked by irregular protrusions, hernias, and indentations (Figs. 2 and 4b–i). These mutant phenotypes were recapitulated when the *Toll-2* alleles were placed over *Df(2R)BSC594*, a deficiency of the region. The CNS phenotype of the *Toll-2* mutants grossly resembles that of *perlecan*, which encodes one of the key components of the CNS basement membrane (Yurchenco 2011), hinting that Toll-2 receptors may function in surface glia to regulate CNS morphology by interfacing with components of the basement membrane. Cell death may also contribute to the observed defects in CNS morphology, as loss of *Toll-2* function has been shown to drive cell death in neurons and neuroblasts (Li *et al*. 2020; Hermanstyne *et al*. 2022).

The *jsZ4* allele represents the sole mutation that we were unable to map to a defined gene (Table 1). Its partially penetrant phenotype is highlighted by an elongated nerve cord and the presence of hernias and protrusions in it (Fig. 2). As we sequenced the genome of this allele at ≥30× coverage, our failure to map its mutant phenotype to a specific gene could arise due to the phenotype being caused by 2 or more mutations, a noncoding mutation, or other reasons.

### Genes that yield an elongated CNS phenotype when mutated

Our largest phenotypic class—an elongated CNS—was identified by 23 mutations and 9 genes. Two of the 9 genes are known tumor suppressors—*lethal giant larvae* (*lgl*) and *brain tumor* (*brat*). We identified a single mutation in *lgl*, a frameshift mutation which when homozygous yields an elongated ventral nerve cord and greatly enlarged brain lobes (Table 1; Fig. 2). Complementation crosses against the *lgl^4^* allele and a deficiency that uncovers *lgl*, *Df(2L)ED50001*, confirmed correspondence between the gene and mutant phenotype. The elongated CNS phenotype likely arises from elevated cell proliferation since *lgl* plays a well-characterized role in restricting cell proliferation (Gateff and Schneiderman 1974). Like *lgl*, loss of *brat* function is known to increase neuroblast and cell proliferation and result in tumorous growths in the brain (Gateff 1994). We identified 3 mutations in *brat* that yielded a moderate CNS elongation phenotype and enlarged brain when homozygous or trans-heterozygous to each other or *Df(2L)Exel8040*, a deficiency of the region (Table 1; Fig. 2).

*worniu*: We identified a nonsense mutation, L117*, in *worniu* that yielded a moderately elongated nerve cord (Table 1; Fig. 2). We confirmed correspondence between *worniu* and the mutant phenotype based on the *worniu*^js1^ allele failing to complement the *worniu^1^* and *worniu^2^* alleles as well as *Df(2L)Exel8034*, which uncovers *worniu*, for the elongated nerve cord phenotype. *worniu* encodes a C2H2-type Zinc finger transcription factor that is expressed in most neuroblasts and has been shown to regulate CNS structure (Ashraf *et al*. 2004).

*Tango1*: We identified a missense mutation, T60I, in the *Tango1* coding region that when homozygous resulted in a moderately elongated CNS (Table 1; Fig. 2). We confirmed correspondence between this mutation and the observed CNS elongation phenotype by complementation against 2 overlapping deficiencies that each uncover *Tango1*, *Df(2L)BSC6*, and *Df(2L)BSC188*. *Tango1* functions at ER exit sites to promote protein secretion and is required to promote the specialized secretion of the large Collagen and Laminin proteins (Saito *et al*. 2009; Malhotra and Erlmann 2011; Wilson *et al*. 2011; Petley-Ragan *et al*. 2016). Thus, the CNS elongation phenotype of *Tango1* likely derives from defective secretion and deposition of basement membrane proteins, as loss of function in the basement membrane proteins, such as *LanB1*, *vkg*, and *Col4a1* lead to an elongated CNS (Pastor-Pareja and Xu 2011; Kim *et al*. 2014; Skeath *et al*. 2017; Dai *et al*. 2018).

**Fig. 5.**
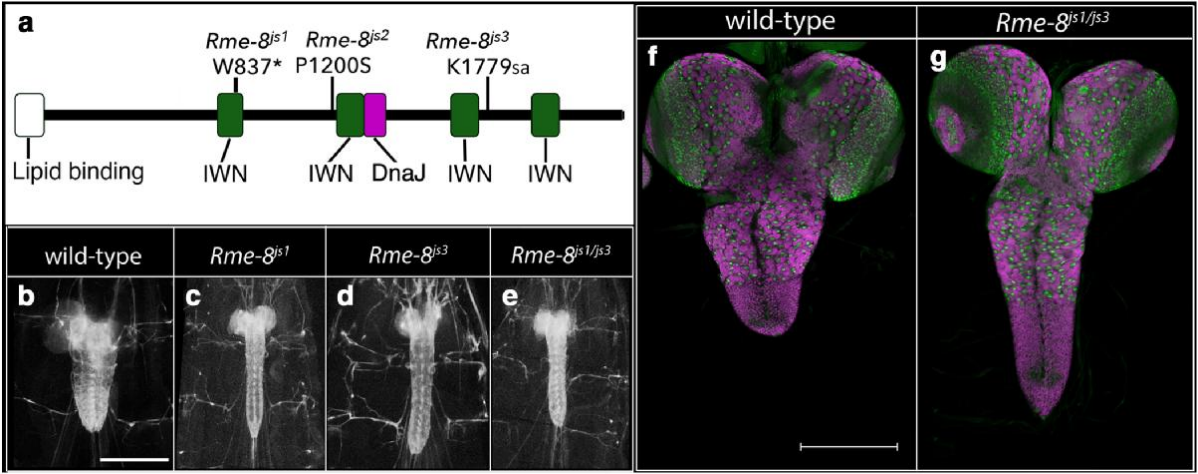
Loss of *Rme-8* function promotes CNS elongation. a) Schematic of Rme-8 protein showing the location of the lipid binding, IWN repeat, and DnaJ domains as well as the location and nature of the 3 identified *Rme-8* alleles. b–e) Ventral views of wild-type or *Rme-8* mutant late-third instar larvae in which 3xP3 RFP expression highlights the CNS. Anterior is up; scale bar is 500 μm. f–g) Ventral views of dissected CNS of wild-type (f) and *Rme-8* mutant (g) late-third instar larvae labeled for neuroblasts with Dpn (green) and neurons with ELAV (magenta). Anterior is up; scale bar is 200 μm.

*Rme-8*: Three mutations in the *Rme-8* gene also gave rise to a moderately elongated CNS phenotype when homozygous or trans-heterozygous to each other (Table 1; Figs. 2 and 5). This phenotype was recapitulated when each mutation was placed over *Df(2R)BSC279*, a deficiency of the region. *Rme-8* encodes a DnaJ domain-containing protein of the Hsp40 chaperone family that also contains 4 IWN repeat (Zhang *et al*. 2001; Norris *et al*. 2022); Rme-8 promotes endocytic recycling of transmembrane proteins, such as Notch (Gomez-Lamarca *et al*. 2015). Using an *Rme-8-T2A-GAL4* CRIMIC insert in the fourth intron of *Rme-8* to drive a UAS-linked nuclear-RFP transgene (Lee *et al*. 2018; He *et al*. 2019), we found that Rme-8 is broadly expressed in the CNS in neurons and glia, with high-level expression in surface glia (Supplementary Fig. 2).

The remaining 5 genes (*Pvf3*, *CG9171*, *C1GalTA*, *ifc*, and *sens-2*) all exhibited severe CNS elongation phenotypes upon loss of their function.

**Fig. 6.**
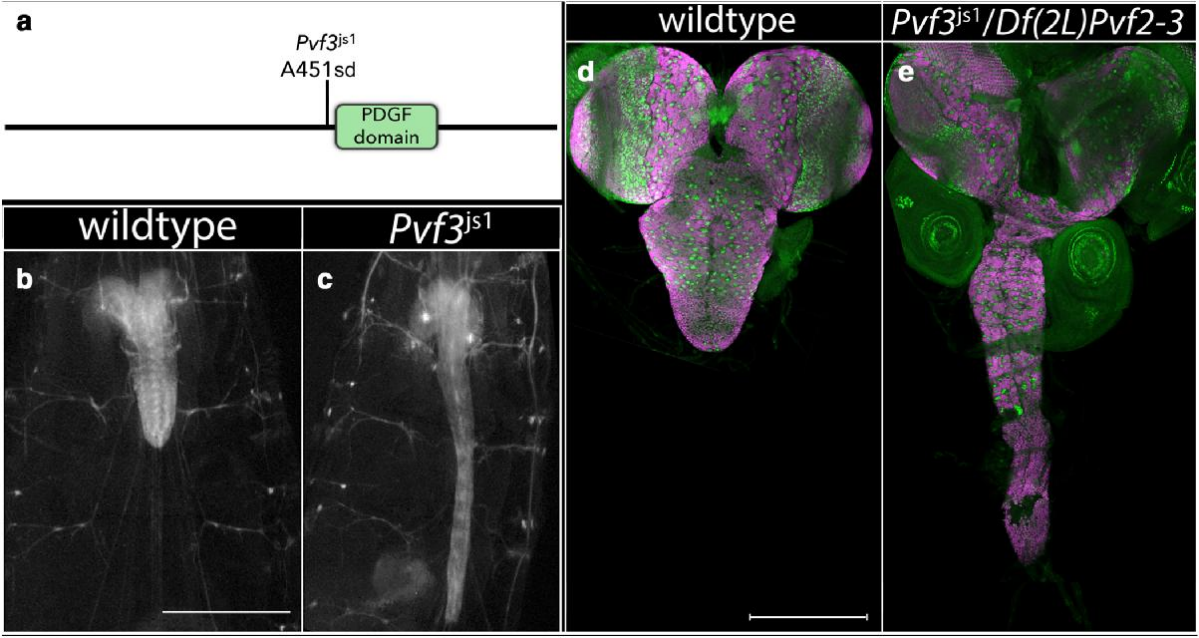
Loss of *Pvf-3* function greatly increases the length of the ventral nerve cord. a) Schematic of Pvf-3 protein with location and nature of the *Pvf-3^js1^* allele. b,c) Ventral views of wild-type or *Pvf-3^js1^* mutant late-third instar larvae in which 3xP3 RFP expression highlights the CNS. Anterior is up; scale bar is 500μm. d,e) Ventral views of dissected CNS of wild-type (d) and *Pvf-3^js1^*/Df(2L)Pvf2–3mutant (e) late-third instar larvae labeled for neuroblasts with Deadpan (Dpn; green) and for neurons with ELAV (magenta). Anterior is up; scale bar is 200 μm.

*Pvf3*: We identified a single mutation in *Pvf3*—a splice site donor mutation just prior to the region that encodes its PDGF domain (Table 1; Fig. 6a). Larvae homozygous for this mutation yield a highly elongated CNS, which is recapitulated when this allele is placed in trans to *Df(2L)ΔPvf2–3*, *Df(2L) BSC291*, or 2 *P* element inserts in *Pvf3* (Fig. 6c–e), confirming correspondence between *Pvf3* and the elongated CNS phenotype. *Pvf3* acts together with *Pvf2* to promote hemocyte survival and migration through activation of the Pvr receptor (Parsons and Foley 2013). Loss of Pvr function or blockade of neural activity has been shown to inhibit nerve cord condensation and promote nerve cord elongation (Olofsson and Page 2005). Our observation that loss of *Pvf3* function alone yields a highly elongated CNS phenotype suggests that *Pvf3* plays a nonredundant role relative to *Pvf2* to promote hemocyte survival, migration, and/or function.

**Fig. 7.**
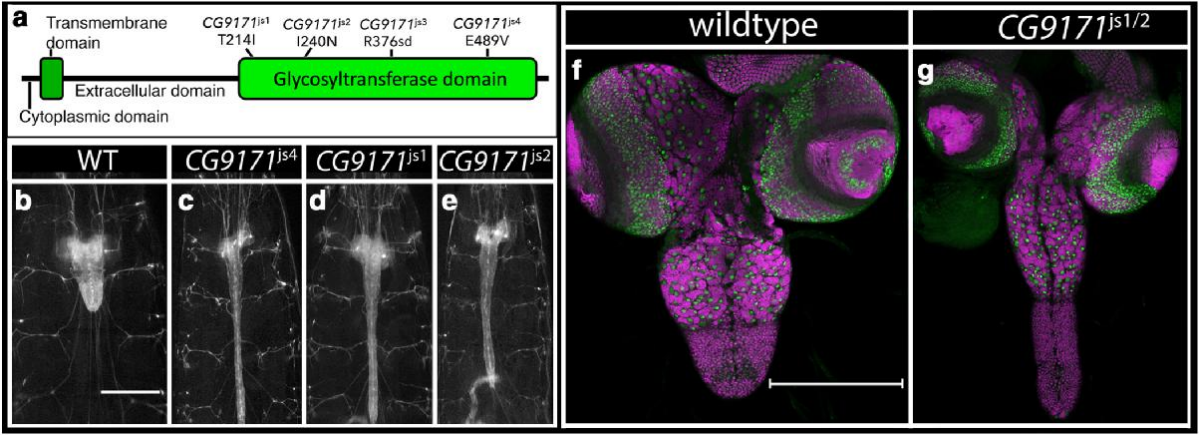
Loss of *CG9171* drives CNS elongation. a) Schematic of CG9171 protein with location and nature of the 4 identified *CG9171* alleles. b–e) Ventral views of wild-type or *CG9171* mutant late-third instar larvae in which 3xP3 RFP expression highlights the CNS. Anterior is up; scale bar is 500 μm. f–g) Ventral views of dissected CNS of wild-type (f) and *CG9171* mutant (g) late-third instar larvae labeled for neuroblasts with Dpn (green) and neurons with ELAV (magenta). Anterior is up; scale bar is 200 μm.

*CG9171*: Four mutant alleles identified *CG9171*, which encodes a predicted glucuronosyltransferase that promotes *O*-linked protein mannosylation. All 4 mutations reside in its extracellular glycosyltransferase domain, likely disrupting enzymatic function (Fig. 7a). Homozygosity or trans-heterozygosity for all possible combinations of the 4 mutations yielded a highly elongated CNS phenotype, but neuroblast formation and neuronal patterning appeared grossly normal (Fig. 7b–g). This phenotype was recapitulated when each allele was placed in trans to *Df(2L)ED334*, a deficiency of the region. The most closely related mammalian homologs of *CG9171*—*B4GAT1* and *LARGE1/2*—act in tandem to drive the *O*-mannosylation of Dystroglycan (Praissman *et al*. 2014). B4GAT1 adds a single glucuronic acid residue onto an Xylose acceptor on Dystroglycan. The related glycosyltransferase LARGE recognizes this epitope and extends it by adding many copies of a repeating disaccharide to form matriglycan. Matriglycan links Dystroglycan to the basement membrane by binding to Laminin and other proteins and plays a key role in disease, as mutations in B4GAT1 in humans cause dystroglycanopathies likely due to the loss of matriglycan on Dystroglycan (Yoshida-Moriguchi *et al*. 2010; Praissman *et al*. 2014; Bigotti and Brancaccio 2021). Our work suggests that defects in the *O*-mannosylation of Dystroglycan and/or other cell surface proteins cause the observed CNS elongation phenotype.

Prior work on the glucuronyltransferase, dGlcAT-P, the fly ortholog of B3GAT1, revealed that it acts in hemocytes to regulate CNS structure (Pandey *et al*. 2011). Loss of *dGlcAT-P* function results in a highly elongated CNS phenotype similar to that observed for mutations in *CG9171*, with additional studies indicating the dGlcAT-P phenotype arises due to mechanical stretching of the CNS (Pandey *et al*. 2011). Forced expression of *dGlcAT-P* in hemocytes, but not in glia or neurons, fully rescued its CNS mutant phenotype, indicating that *dGlcAT-P* acts in hemocytes to govern CNS structure. These results support the idea that CG9171 acts in hemocytes to control CNS structure; expression studies support this idea, as they show strong CG9171 expression in hemocytes (Ozturk-Colak *et al*. 2024).

**Fig. 8.**
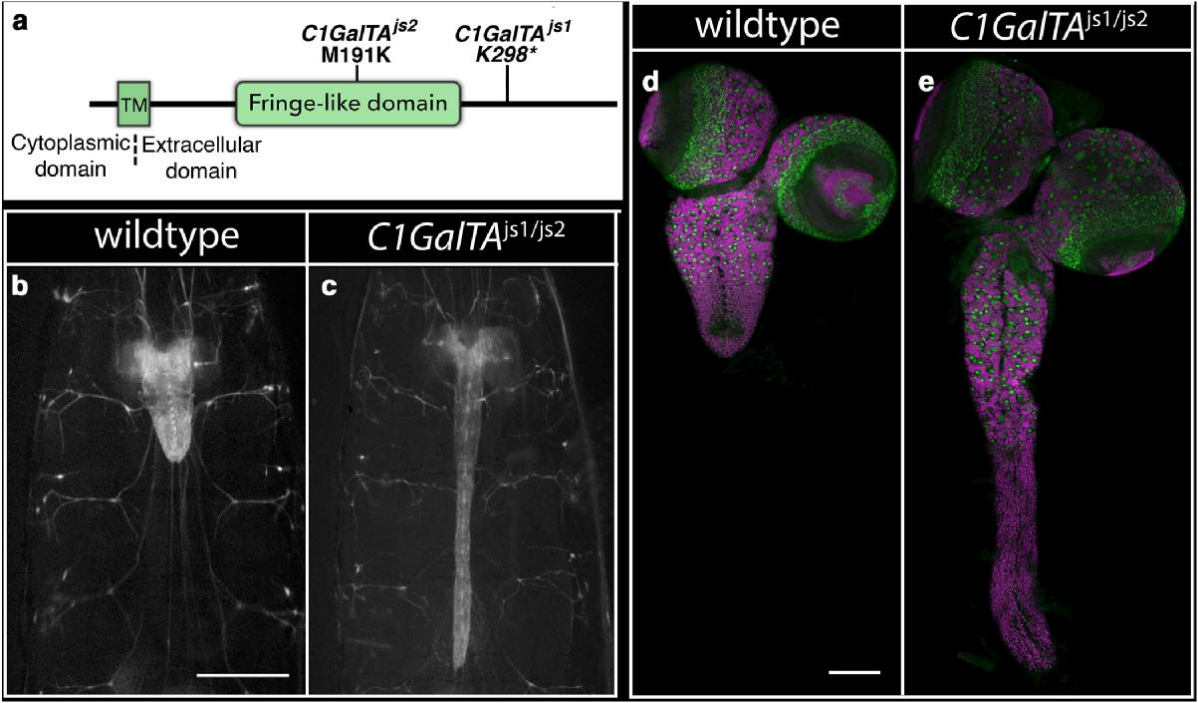
Loss of *dC1GalTA* function promotes CNS elongation. a) Schematic of C1GalTA protein with the location of its Fringe-like glycosyltransferase domain and the nature of the 2 identified alleles. b,c) Ventral views of wild-type or *C1GalTA^js1/js2^* mutant late-third instar larvae in which 3xP3 RFP expression highlights the CNS. Anterior is up; scale bar is 500 μm. d,e) Ventral views of dissected CNS of wild-type (d) and *C1GalTA^js1/js2^* mutant (e) late-third instar larvae labeled for neuroblasts with Deadpan (Dpn; green) and for neurons with ELAV (magenta). Anterior is up; scale bar is 100 μm.

*C1GalTA*: Like *CG9171*, the 2 alleles in *Core 1 Galactosyltransferase A* (*C1GalTA; CG9520*) yielded a highly elongated CNS phenotype (Table 1; Figs. 2 and 8), which was recapitulated when each allele was placed over a deficiency of the region, *Df(2L)BSC204*. Unlike the CG9171 phenotype in which the mutant ventral nerve cord is supple and flexible, *C1GalTA* mutant larvae exhibited a highly elongated, very brittle nerve cord and flat and misshapen brain lobes (Fig. 8b–e). These phenotypes are most obvious in larvae homozygous for the phenotypically stronger *C1GalTA^js1^* allele. C1GalTA promotes protein glycosylation by adding galactose in a β1,3 linkage to *N*-acetylgalactosamine (GalNAc) on proteins. In flies, a prior study showed that *dC1GalTA* is required for glycosylation of Laminin and the presence of T antigen (Gal β1,3 GalNAc) on hemocytes, and that loss of *C1GalTA* function drives CNS elongation (Lin *et al*. 2008).

**Fig. 9.**
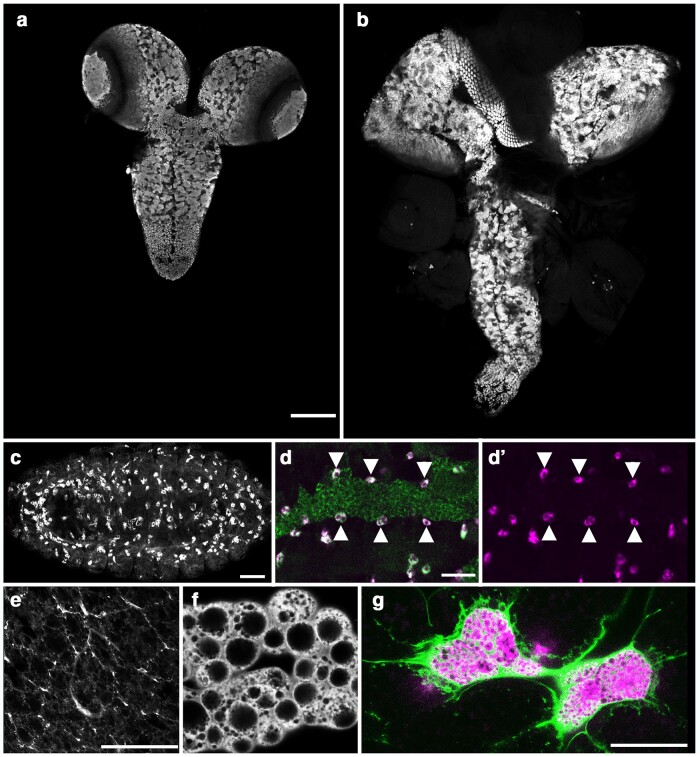
*Papilin* regulates CNS structure and is sufficient to drive the accumulation of Vkg-GFP in the fat body. a,b) Ventral view of CNS of wild-type (a) and *ppn^MI03189^* mutant late-third instar larvae (b) labeled for ELAV. Anterior is up; scale bar is 100 μm. c,d) Stage 15–16 embryos labeled for Ppn (gray, c; magenta, d–d′) and Collagen 4a1 (green, d). Anterior is to the left; scale bars are 50 μm. Arrowheads in d–d′ point to Ppn^+^ hemocytes immediately adjacent to the fat body. e–g) Vkg-GFP localization (gray in e, f; green in g) in wild-type fat body (e), fat body in which Ppn[RG] is expressed in all fat body cells (f), and fat body in which small cell clones, marked in magenta, ectopically express Papilin protein (g). Scale bar is 50 μm for panels e and f and 25 for panel g.

The *C1GalTA* mutant phenotype resembles that of *papilin* (*ppn*), a third chromosomal gene that we were working on in parallel to the genetic screen due to its sequence similarity to AdamTS genes (Campbell *et al*. 1987; Kramerova *et al*. 2000; Keeley *et al*. 2020). Ppn is a key component of the basement membrane, where it has been shown to facilitate Collagen IV removal to promote basement membrane remodeling (Keeley *et al*. 2020). Like the *C1GalTA* mutant phenotype, the reduction of *ppn* function yields a highly elongated and brittle nerve cord and flat, misshapen brain lobes (Fig. 9a,b). Ppn-specific antibodies showed that *ppn* is expressed in hemocytes, the cells that assemble and disassemble basement membranes, but not in most other tissues (Fig. 9c,d), including the fat body—the major producer of basement membrane proteins in larvae (Pastor-Pareja and Xu 2011). We hypothesize that hemocytes deposit Ppn on most tissues during development.

Most basement membrane components, like Col4a1, Vkg, Nidogen, and Perlecan, are produced by fat body cells (Pastor-Pareja and Xu 2011), but Ppn is not expressed in the fat body, suggesting there may be a physiological reason for the exclusion of Ppn expression in the fat body. To test this model, we misexpressed *ppn* in the fat body and asked if it altered basement membrane protein localization by visualizing the localization of a GFP protein trap in Viking (Morin *et al*. 2001; Buszczak *et al*. 2007). Upon forced *ppn* expression in the fat body, we observed a massive, cell-autonomous retention of Viking-GFP in fat body cells (Fig. 9e–g), demonstrating the incompatibility of fat body *ppn* expression with appropriate Viking secretion and suggesting that Ppn physically associates with Viking in vivo to help mediate the removal of the basement membrane components. The similarity of the *ppn* and *C1GalTA* mutant phenotypes suggests that C1GalTA acts with Ppn to promote Viking/Collagen IV removal from the basement membrane and that failure to do so results in a brittle, elongated CNS. Future work that determines how these genes functionally interface with each other should help clarify the molecular basis of basement membrane remodeling and the control of tissue structure.

*infertile* crescent: Three alleles identified *infertile crescent* (*ifc*; Table 1), which encodes the sole *Drosophila* dihydroceramide desaturase that converts dihydroceramide to ceramide in the last step of the de novo ceramide biosynthesis pathway (Jung *et al*. 2017; Hannun and Obeid 2018). Loss of *ifc* results in an elongated CNS marked by bulges in peripheral nerves (Fig. 2); we characterized the function of *ifc* in detail in a separate study and found it functions primarily in glia to promote glial homeostasis and to guard against neuronal cell death (Zhu *et al*. 2024).

### Senseless-2 acts in perineurial glia to regulate CNS structure

**Fig. 10.**
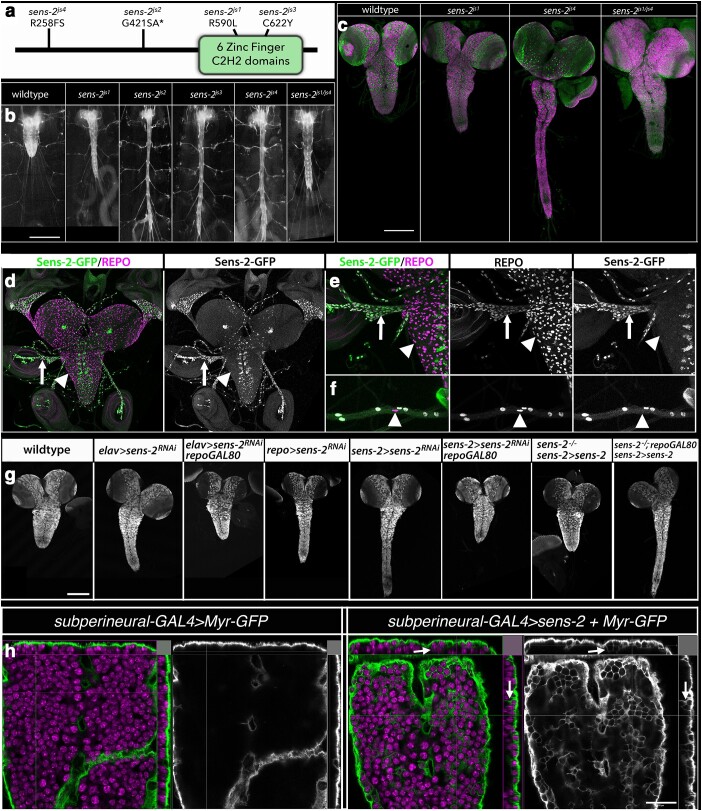
*sens-2* acts in peripheral glia to regulate CNS structure. a) Schematic of Sens-2 protein with location and nature of the 4 identified *sens-2* mutations. b) Ventral views of late-third instar larvae of indicated genotype showing 3xP3 RFP expression in the CNS and nerves. Anterior is up; scale bar is 500 μm. c) Ventral views of photomontages of late-third instar larvae of indicated genotypes labeled for neuroblasts with Deadpan (Dpn; green) and for neurons with ELAV (magenta). Anterior is up; scale bar is 200 μm. d–f) Ventral views of low (d) and high magnification (e, f) images of the CNS (d, e) and peripheral nerves (f) of late-third instar larvae labeled for *sens-2-GFP* (green and grayscale) to mark *sens-2* expressing cells and REPO (magenta) to mark glia. Arrows in d–e identify peripheral glia that express *sens-2-GFP*; arrowheads identify central glia that lack GFP expression. Arrowhead in f marks *sens-2-GFP*-negative wrapping glia in peripheral nerve. g) Ventral views of the dissected CNS from late-third instar larvae of indicated genotype labeled for ELAV to mark neurons and highlight the structure of the CNS. Anterior is up; scale bar is 200 μm. h) Ventral view and Y–Z and X–Z projection views of late-third instar larvae of indicated genotype labeled for Myr-GFP (green/grayscale) and ELAV to label neurons (magenta). Arrows point to Myr-GFP + membrane invaginations that enwrap neurons. Anterior is up; scale bar is 20 μm.

Four mutant alleles identified the *senseless-2* (*sens-2*) gene, which encodes a member of the Zinc finger C2H2 superfamily of proteins and possesses 6 C2H2 Zinc finger domains (Figs. 2 and 10a). Three of the alleles—*sens-2^js2^*, *sens-2^js3^*, and *sens-2^js4^*—introduce, respectively, an early splice site mutation, a missense mutation in a conserved cysteine in the Zinc finger domain, and an early frameshift mutation (Table 1). These alleles when homozygous or trans-heterozygous to each other or a deficiency of the region, *Df(2L)ED6569*, yielded an essentially identical highly elongated CNS phenotype (Fig. 10b,c) and are likely null or strong hypomorphic alleles. The fourth allele, *sens-2^js1^*, yields a modest CNS elongation phenotype; larvae trans-heterozygous for *sens-2^js1^* and *sens-2^js4^* exhibit an intermediate CNS elongation phenotype between the 2 alleles, identifying *sens-2^js1^*, which contains a missense mutation in the zinc finger domain (Table 1; Fig. 10a), as a weak hypomorphic allele of *sens-2*.

The *sens-2* CNS elongation phenotype manifests in first instar larvae and is maintained throughout larval life and into the pupal stage at which point *sens-2* homozygous flies die. In live third instar larvae, the CNS appears to be under tension from both laterally and posteriorly projecting peripheral nerves, with the posterior nerves being shorter in length in *sens-2* mutant larvae relative to wild-type (Figs. 2 and 10b). The overall shape of the CNS is dramatically altered, but the overall pattern and number of neuroblasts and neurons appeared grossly normal (Fig. 10c).

To clarify the cellular basis for the *sens-2* CNS elongation phenotype, we tracked *sens-2* expression in larvae by generating a Sens-2 specific antibody and a *sens-2-T2A-GAL4* CRIMIC line (*sens-2-GAL4*) and leveraging a *sens-2-GFP* transgene surrounded by its endogenous genomic locus (Kudron *et al*. 2018; see Materials and Methods). These tools revealed that *sens-2* is expressed in subsets of glia and neurons in the larval nervous system (Fig. 10d–f; Supplementary Figs. 3 and 4). We focused on *sens-2* expression in the nervous system, as its function in glia, neurons, or both likely contributes to the observed CNS phenotype. Double-labeling experiments with REPO to mark all glia and ELAV to label all neurons revealed that *sens-2* is expressed in a small number of CNS neurons and most glia in peripheral nerves, but that *sens-2* is not detected in any glia within the CNS itself (Fig. 10d,e; Supplementary Figs. 3 and 4). Recent scRNA analysis of the larval CNS supports the restriction of *sens-2* expression to surface glia and subsets of neurons (Nguyen *et al*. 2024). More detailed analysis of *sens-2* expression within peripheral nerves of late-third instar larvae suggested that *sens-2* is expressed in all perineurial glia and weakly in some subperineural glia, but that its expression appears to be excluded from wrapping glia and some subperineurial glia (Fig. 10f; Supplementary Figs. 3 and 4). We conclude that *sens-2* expression demarcates PNS perineurial glia (*sens-2*-positive) from CNS perineurial glia (*sens-2*-negative), identifying *sens-2* as one of few markers to distinguish a glial subtype along the CNS–PNS axis.

To determine if *sens-2* function is required in glia, neurons, or both to regulate CNS structure, we used the GAL4/UAS system together with either a *UAS-sens-2-RNAi* transgene or a *UAS-sens-2* transgene (Fig. 10g). Depletion of *sens-2* function specifically in neurons via RNAi using *elav-GAL4* together with *repo*-*GAL80* had no effect on CNS structure or morphology (*n* = 11/11; Fig. 10g), but depleting *sens-2* function specifically in glia using *repo-GAL4* recapitulated the elongated nerve cord phenotype observed in *sens-2* mutant larvae (*n* = 11/11; Fig. 10g). We note that using *elav-GAL4* in the absence of *repo-GAL80* resulted in elongated nerve cords (*n* = 11/12) due to the ability of *elav-GAL4* to drive significant gene expression in glia (Berger *et al*. 2007). We observed similar results with the *sens-2-GAL4* line: RNAi-mediated depletion of *sens-2* in all *sens-2* expressing cells elicited an elongated nerve cord (*n* = 12/12), but when *repo-GAL80* was used to block *sens-2* RNAi in glia, the phenotype disappeared, and nerve cords appeared wild-type (*n* = 11/11). Gene rescue experiments corroborated the above results (Fig. 10g): Expression of a wild-type *sens-2* transgene under control of the *sens-2-GAL4* line in otherwise *sens-2* mutant larvae fully rescued the *sens-2* CNS phenotype (*n* = 10/10), but when *repo-GAL80* was used to block transgene expression specifically in glia, the *sens-2* elongated CNS phenotype reappeared (*n* = 10/10). We conclude that *sens-2* function is required solely in PNS perineurial and perhaps subperineural glia to control CNS structure, and that loss of *sens-2* function in glial cells alone is sufficient to yield the observed elongated nerve cord phenotype.

To assess the function of *sens-2* in different glial subtypes, we leveraged the GAL4/UAS system and GAL4 lines specific for each glial subtype to drive *sens-2* expression and that of *Myr-GFP*, which outlines cell morphology, in each glial subtype. We observed no gross change in cell morphology upon *sens-2* misexpression in astrocyte-like, cortex, and ensheathing glia. Forced expression of *sens-2* by 2 different perineurial-specific GAL4 lines, which also drive strong gene expression in the gut, resulted in early larval lethality inhibiting our ability to assess the impact of *sens-2* misexpression in this glial subtype. Misexpression of *sens-2* in subperineural glia, however, drove a clear change in cell morphology (Fig. 10h). Subperineural glia normally form a thin, flat cell layer that fully encircles the circumference of the CNS and peripheral nerves and resides immediately interior to the perineurial glial cell layer (Yildirim *et al*. 2019). Upon *sens-2* misexpression in subperineural glia, these glial cells still fully encircle the CNS, but they now also extend cell membranes into the interior of the CNS to fully or partially enwrap individual neurons (arrows, Fig. 10h). *sens-2* misexpression in subperineural glia then modifies the behavior of this glial cell type, bestowing on it the ability to enwrap neuronal cell bodies in addition to the entire CNS, suggesting that *sens-2* alters the membrane properties of surface glia to facilitate their ability to wrap peripheral nerves.

Our work on *sens-2* suggests that it acts as a genetic switch to distinguish the functional properties of surface glia found In the PNS from those found in the CNS. A key difference between these 2 tissues is their diameter—peripheral nerves are tiny in diameter relative to the much larger CNS. In this context, the ability of forced *sens-2* expression to alter the behavior of subperineural glia so that they inappropriately enwrap adjacent neuronal cell bodies highlights a profound impact of *sens-2* expression on key functional properties of surface glia—their ability to enwrap (or not enwrap) small diameter structures, such as cells or nerves. Our work did not clarify the molecular basis through which *sens-2* dictates such functional properties. In the future, it will be important to identify the transcriptional targets of *sens-2* to clarify the exact mechanism through which it governs the development and differentiation of surface glia in the PNS.

Summary: Prior work from many laboratories has highlighted the importance of interactions between the basement membrane and glial cells in dictating CNS structure (Kim *et al*. 2014; Meyer *et al*. 2014; Skeath *et al*. 2017). Initial results from our screen reinforce these findings. *Tango1*, *C1GalTA*, *ppn*, and *pvf3* all appear to act on basement membrane proteins, form components of the basement membrane, or regulate the survival or migration of hemocytes, the bricklayers, and tuckpointers of the basement membrane. Continued work on these genes, especially *C1GalTA* and *ppn*, which likely act in the same pathway, holds the promise of clarifying our understanding of basement membrane function and remodeling on tissue structure. Conversely, *sens-2* and *ifc* act in glial cells to regulate CNS structure (this paper; Zhu *et al*. 2024). We expect *Rme-8* and *Toll-2* also act in glia to regulate CNS shape, with future work needed to confirm this prediction and to clarify if and how such factors interface with the basement membrane to govern CNS morphology.

## Supplementary Material

jkae152_Supplementary_Data

## Data Availability

Strains and antibodies are available upon request; many of the fly strains generated for this study will be deposited at the Bloomington Drosophila Stock Center. Whole genome sequencing data are available at Genbank under the NCBI accession number: BioProject PRJNA1128589; this information can also be found in Supplementary File 1. The authors affirm that all data necessary for confirming the conclusions of the article are present within the article, figures, and tables. Supplemental material available at G3 online.
