# Supplementary material for: A genetic screen in Drosophila uncovers a role for senseless-2 in surface glia in the peripheral nervous system to regulate CNS morphology
Source: G3 (Bethesda). 2024 Jul 12;14(9):jkae152. doi: 10.1093/g3journal/jkae152 (PMC11373656; doi:10.1093/g3journal/jkae152)
Supplement: jkae152_Supplementary_Data [file jkae152_supplementary_data.zip › File_S1_G3-2024-405157.docx]

**Figure S1:**


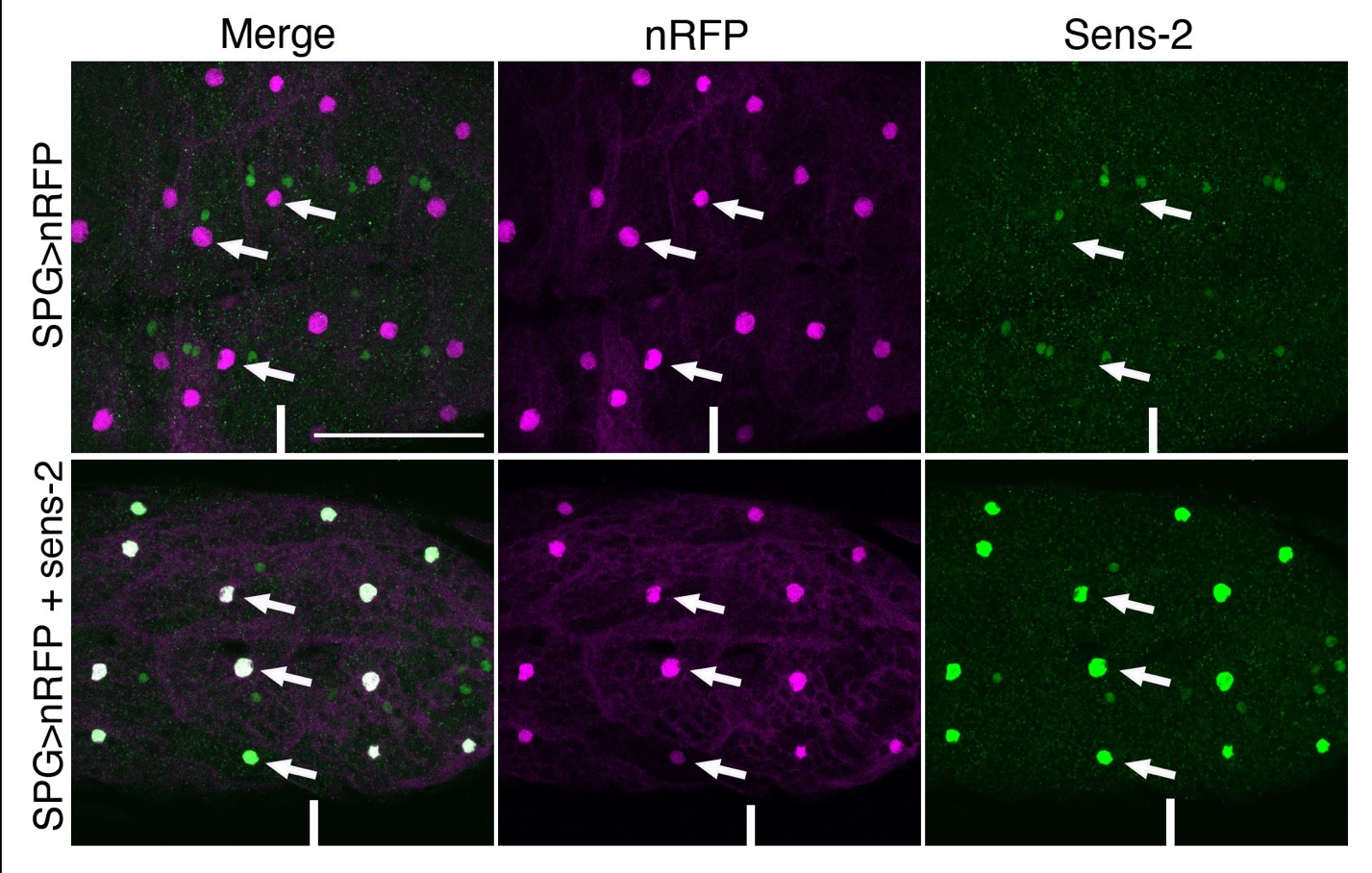


**Figure S1)** Senseless-2 antibody recognizes ectopically expressed Senseless-2 protein. High magnification views of the abdominal region of the ventral nerve cord from late third instar larvae expressing either nuclear-RFP (nRFP; top) or nRFP and senseless-2 (bottom) under the control of the subperineural glia specific GAL4 line GMR54C07-GAL4. Top: The senseless-2 antibody does not detect Senseless-2 protein in subperineural glia in otherwise wild-type larvae that express nRFP in subperineural glia (arrows). Bottom: The Senseless-2 antibody strongly detects Senseless-2 protein in subperineurial glia upon GAL4-mediated expression of *senseless-2* in these cells (arrows). Line represents midline of ventral nerve cord. Anterior is up; scale bar is 50 microns.

**Figure S2:**


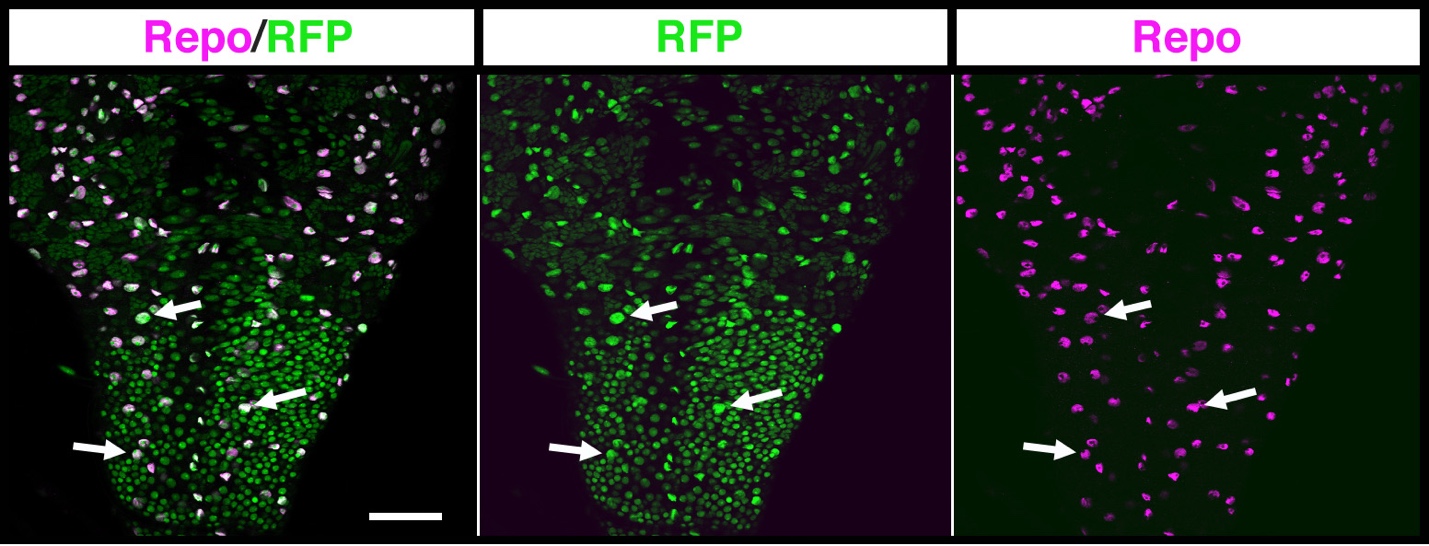


**Figure S2**) Rme-8 is expressed in glia and neurons. Ventral view of *Rme-8^CR00765^-GAL4/UAS-nuclear-RFP* late third instar larvae showing REPO expression (magenta) and endogenous RFP labeling (green). Arrows highlight surface glia, which exhibit higher level of RFP expression than surrounding neurons.

**Figure S3:**


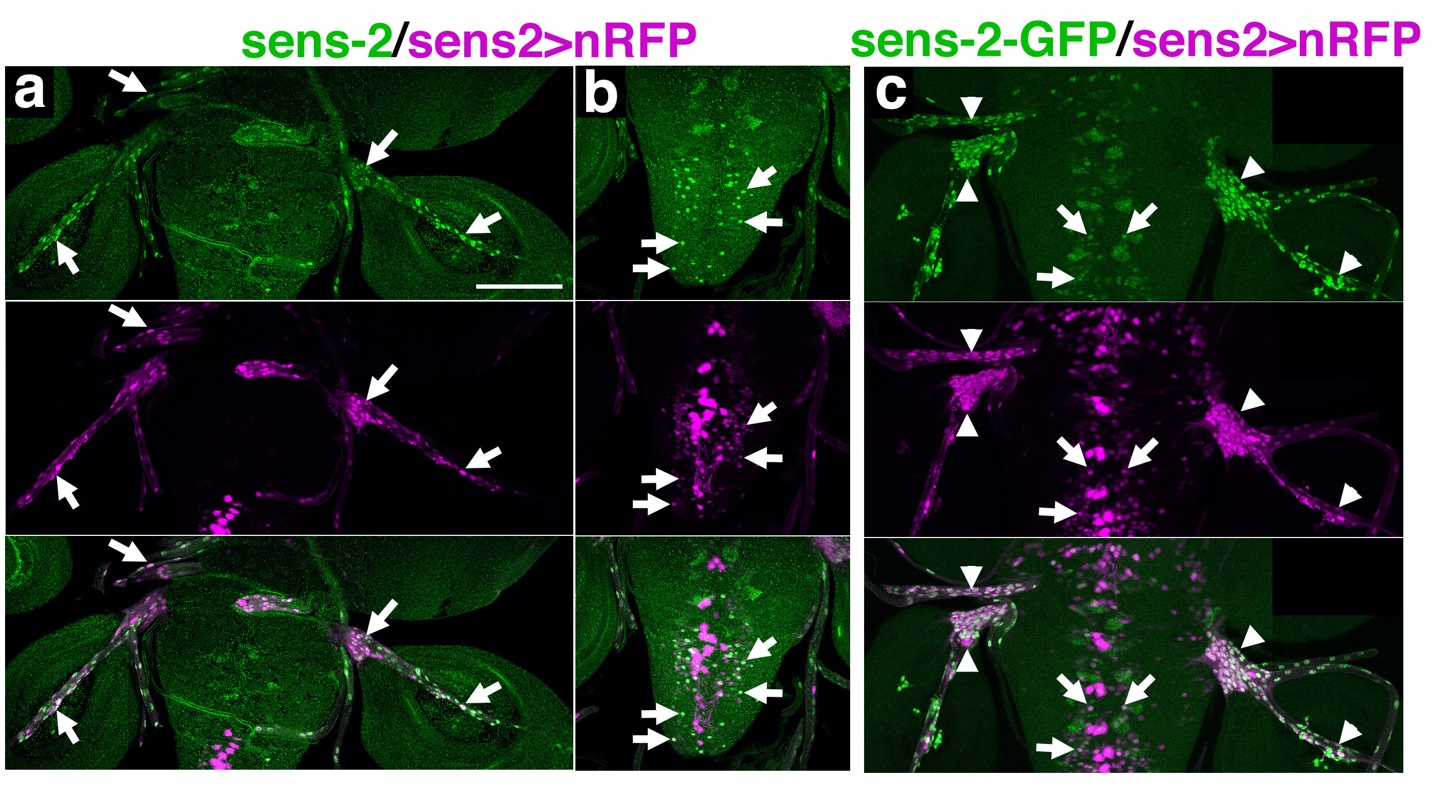


**Figure S3)** Coincident expression of sens-2 protein, sens-2-T2A-GAL4 CRIMIC line, and sens-2-GFP line.

a-b) Ventral views of CNS and peripheral nerves of late third instar *sens-2-T2A-GAL4>nRFP* larvae labeled with sens-2 antibody (green) and nRFP (red) highlighting *sens-2* expression in peripheral nerves (a) and within the CNS (b). Arrows in panel a highlight co-expression of Sens-2 and nRFP in peripheral glia; arrows in panel b highlight co-expression of sens-2 and nRFP in neurons. c) Ventral views of CNS and peripheral nerves of late third instar *sens-2-T2A-GAL4>nRFP* marked for GFP and nRFP. Arrows in panel c highlight co-expression in peripheral glia; arrowheads identify co-expression in neurons. Anterior is up; scale bar is 100 μm.

**Figure S4:**

**
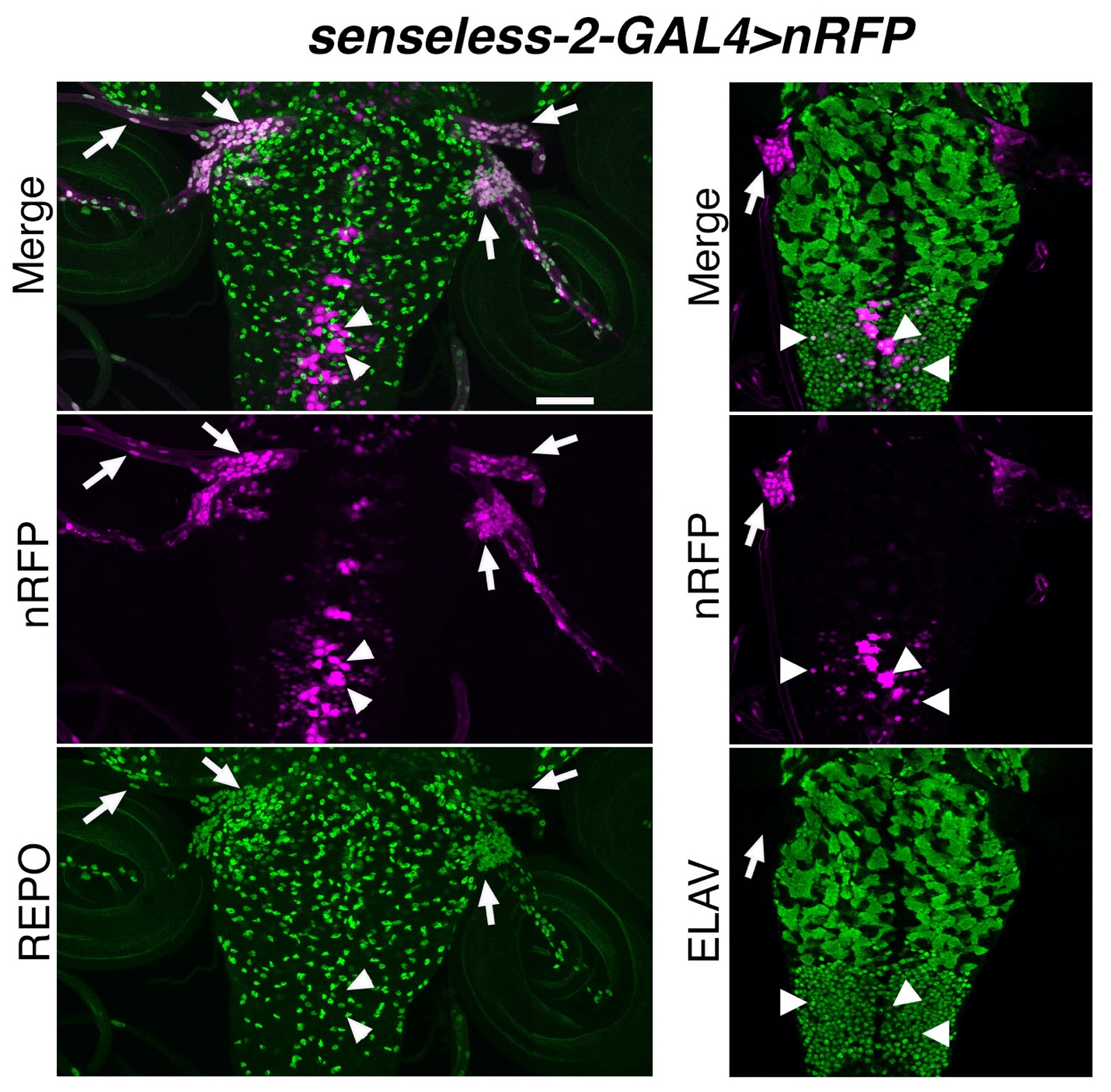
**

**Figure S4**) *sens-2-GAL4* labels neurons in the CNS and peripheral glia. Ventral views of the CNS and peripheral nerves of late third instar *sens-2-GAL4>nRFP* larvae labeled for RFP and repo to mark glia (green; left) or ELAV to mark neurons (green; right). Left) Expression of nRFP (red) is co-expressed with REPO (green) in peripheral glia, but not in any cells in the CNS. Right) Expression of nRFP (red) is co-expressed with ELAV (green) in neurons in the CNS (arrowheads). Arrows point to peripheral glia; arrowheads to sens-2-positive neurons. Anterior is up; scale bar is 100 μm.

Genbank accession information for whole genome sequence information: BioProject ID PRJNA1128589

**Table S1) List of fly stocks used in this paper (see also reagents table):**

| Genotype | Source | Notes |
| --- | --- | --- |
| *M{3xP3-RFP.attP}ZH-51D* | Derived from BDSC #24483 | Wildtype. Control for 3xP3 background. |
| P[w+] lines used to map genes on the genetic map |  |  |
| *w[1118]; P{y[+mDint2] w[BR.E.BR]=SUPor-P}KG07698/CyO (Genetic map position: 2-13)* | BDSC #15116 |  |
| *y[1] w[67c23]; P{y[+mDint2] w[BR.E.BR]=SUPor-P}grp[KG08033] (Genetic map position: 2-52)* | BDSC #14931 |  |
| *y[1] w[67c23]; P{y[+mDint2] w[BR.E.BR]=SUPor-P}whd[KG01596] (Genetic map position: 2-62)* | BDSC #13731 |  |
| *y[1] w[67c23]; P{y[+mDint2] w[BR.E.BR]=SUPor-P}KG07930 (Genetic map position: 2-86)* | BDSC #14672 |  |
|  |  |  |
| Lines used for complementation tests |  |  |
| *LanB1^KG03456^/CyO* | BDSC #13907 |  |
| *Df(2L)BSC172/CyO* | BDSC #9605 | Small deficiency that removes *viking* and Col4a1 |
| *dia[5]/CyO* | BDSC #9138 | Failed to complement *dia^js1^* |
| *Df(2L)BSC6/CyO* | BDSC #6338 | Failed to complement *Tango1^js1^* |
| *Df(2L)BSC188/CyO* | BDSC #9615 | Failed to complement *Tango1^js1^* |
| *l(2)lgl^4^/CyO* | BDSC #36289 | Failed to complement *l(2)lgl^js1^* |
| *Df(2L)ED50001/CyO* | BDSC #24626 | Failed to complement *l(2)lgl^js1^* |
| *worniu^1^/CyO* | BDSC #3155 | Failed to complement *wor^js1^* |
| *worniu^2^/CyO* | BDSC #25170 | Failed to complement *wor^js1^* |
| *Df(2L)ΔPvf2-3/CyO* | Parsons and Foley, 2013 | Failed to complement *Pvf3^js1^* |
| *Pvf3[MI04168]/CyO* | BDSC #37270 | Failed to complement *Pvf3^js1^* |
| *Pvf3[MI15271]/CyO* | BDSC #61008 | Failed to complement *Pvf3^js1^* |
| *P{EP}PpnG4259/CyO* | BDSC #31782 |  |
| *ppn^MI03189^/CyO* | BDSC #36216 |  |
| *Df(2L)ED334/CyO* | BDSC #9343 | Fails to complement CG9171 |
| *w[1118]; P{y[+t7.7] w[+mC]=GMR78D01-GAL4}attP2* | BDSC #47014 | Drives GAL4 in fat body |
| *w, hs-flp* | BDSC #6 |  |
| *P{w[+mC]=GAL4-Act5C(FRT.CD2).P}S, P{w[+mC]=UAS-RFP.W}3, P{w[+mC]=UAS-Ppn-RG}/TM6, Tb[1]* | Derived from BDSC #30558 |  |
|  |  |  |
| *y[1] sc[*] v[1] sev[21]; P{y[+t7.7] v[+t1.8]=TRiP.HMS01394}attP2* | BDSC #34984 | *UAS-sens-2-RNAi* |
| *PBac{sens-2-GFP.FPTB}VK00031* | BDSC #93096 | *sens-2-GFP* |
|  |  |  |
| *w[1118]; P{y[+t7.7] w[+mC]=GMR54H02-GAL4}attP2* | BDSC #45784 | Cortex glia GMR-GAL4 |
| *w[1118]; P{y[+t7.7] w[+mC]=GMR86E01-GAL4}attP2* | BDSC #45914 | Astrocyte-like glia GMR-GAL4 |
| *w[1118]; P{y[+t7.7] w[+mC]=GMR56F03-GAL4}attP2* | BDSC #39157 | Ensheathing glia GMR-GAL4 |
| *w[1118]; P{y[+t7.7] w[+mC]=GMR54C07-GAL4}attP2* | BDSC #50472 | Subperineurial glia GMR-GAL4 |
| *w[1118]; P{y[+t7.7] w[+mC]=GMR85G01-GAL4}attP2* | BDSC #40436 | Perineurial glia GMR-GAL4 |
| *P{w[+mC]=GAL4-repo/TM6 Tb* | BDSC #7415 | Pan-glial driver |
| *P{w[+mC]=GAL4-elav[C155]}* | BDSC #458 | Pan-neuronal driver; also expressed in glia |
| *P{w[+mC]=GAL4-elav[C155]}; Repo-GAL80/TM6, Tb* | Skeath et al., 2017 | Pan-neuronal driver |
| *TI[CRIMIC.TG4.1}Rme-8^CR00765^-TG4.1/SM6a* | BDSC #80650 | T2A-GAL4 insert  in *Rme-8* |
| *P{y[+t7.7] v[+t1.8]=UAS-TransTimer.v+}attP2* | BDSC #93411 | Contains nuclear targeted UAS-TagRFP transgene |
|  |  |  |
| *sens-2-GAL4; CyO Tb; repo-GAL4/TM6 Tb* | This paper |  |
| *sens-2^js4^/CyO Tb; UAS-sens-2* | This paper |  |
